# CCR2 Signaling Promotes Brain Infiltration of Inflammatory Monocytes and Contributes to Neuropathology during Cryptococcal Meningoencephalitis

**DOI:** 10.1128/mBio.01076-21

**Published:** 2021-07-27

**Authors:** Jintao Xu, Anutosh Ganguly, Jessica Zhao, Michel Ivey, Rafael Lopez, John J. Osterholzer, Clifford S. Cho, Michal A. Olszewski

**Affiliations:** a Research Service, Ann Arbor VA Health System, Department of Veterans Affairs Health System, Ann Arbor, Michigan, USA; b Division of Pulmonary and Critical Care Medicine, Department of Internal Medicine, University of Michigan Health System, Ann Arbor, Michigan, USA; c Division of Hepatopancreatobiliary and Advanced Gastrointestinal Surgery, Department of Surgery, University of Michigan, Ann Arbor, Michigan, USA; University of Massachusetts Medical School; Texas Christian University

**Keywords:** CCR2, cryptococcal meningoencephalitis, central nervous system infections, immunopathogenesis, inflammatory monocyte

## Abstract

Cryptococcal meningoencephalitis (CM) is a leading cause of central nervous system (CNS) infection-related mortality worldwide, with surviving patients often developing neurological deficiencies. While CNS inflammation has been implicated in the pathogenesis of CM, little is known about the relative contribution of the specific inflammatory/immune pathways to CNS pathology versus fungal clearance. Increased cerebrospinal fluid level of C-C chemokine receptor 2 (CCR2) ligand CCL2 is associated with disease deterioration in patients with CM. Using a murine model, we investigated the role of the CCR2 pathway in the development of CNS inflammation and pathology during CM. We found that CCR2-deficient mice exhibited improved 28-day survival and alleviated neurological disease scores despite a brain fungal burden higher than that of the WT mice. Reduced CM pathology in CCR2-deficient mice was accompanied by markedly decreased neuronal cell death around cryptococcal microcysts and restored expression of genes involved in neurotransmission, connectivity, and neuronal cell structure in the brains. Results show that CCR2 axis is the major pathway recruiting CD45^hi^CD11b^+^Ly6C^+^ inflammatory monocyte to the brain and indirectly modulates the accumulation of CD4^+^ T cells and CD8^+^ T cells. In particular, CCR2 axis promotes recruitment of interferon gamma (IFN-γ)-producing CD4^+^ T cells and classical activation of myeloid cells. In this context, CCR2 deletion limits the immune network dysregulation we see in CM and attenuates neuropathology. Thus, the CCR2 axis is a potential target for interventions aimed to limit inflammatory CNS pathology in CM patients.

## INTRODUCTION

The fungal pathogen Cryptococcus neoformans is a common cause of adult meningoencephalitis in many parts of the world, resulting in nearly 200,000 deaths worldwide each year (1, 2). Cryptococcal meningoencephalitis (CM) is not only characterized by a high mortality rate (up to 30 to 50%) (3, 4), but survivors frequently develop long-lasting severe neurological sequelae, including memory loss, vision deficiencies, hearing and speech impairments, and motor deficits (2, 5). Recent studies revealed that inflammation triggered in response to *Cryptococcus* rather than the pathogen itself can lead to neuropathology in a subset of HIV^+^ and HIV^−^ CM patients (2, 6–9). Thus, selective control of the pathological immune pathways may become an important element of therapies aimed at preventing the immune-mediated damage within the CNS.

While required for anticryptococcal defenses, CD4^+^ T cells have emerged as critical drivers of immunopathology in both human and murine CM (10–13). Rapid reconstitution of T cells in HIV^+^ CM patients by antiretroviral therapy (ART) can lead to immune reconstitution inflammatory syndrome (c-IRIS), characterized by a dysregulated T cell response (14). Likewise, noncompromised CM patients often develop severe neurological pathology that is steroid responsive (6). CD4^+^ T cells also orchestrate lethal immune pathology in animal models (12, 13, 15). However, CD4^+^ cells do not perform their immune functions on their own but require antigen-presenting cellular partners to become activated and to execute their effector functions in neuroinflammatory diseases (16). Myeloid cells, especially recruited monocytes and monocyte-derived cells, exacerbate pathological T cell responses, for example, by secreting chemokines to recruit T cells and by stimulating their antigen-specific cytokine production (16–18). At the same time, upon activation by the T cells, these cells can produce cytotoxic molecules, such as reactive oxygen species known to cause collateral tissue damage (17, 18). CD45^high^Ly6C^+^CD11b^+^ inflammatory monocytes (IM) are recruited in the CNS during CM (13, 19, 20), but their function and mechanisms responsible for their recruitment remain unclear.

CC chemokine receptor 2 (CCR2) is the major chemokine receptor required for monocyte migration from bone marrow to the inflammatory sites through binding CC chemokine ligands such as CCL2 (21). The role of CCR2 signaling in pulmonary cryptococcal infections is predominantly protective by promoting protective Th1 antifungal immunity over nonprotective Th2 (22–25). However, in already Th2-biased response to C. neoformans, CCR2^+^ IM can mediate pathological effects in the lungs (26). Specific roles of the CCR2 pathway remain less defined in the CNS during CM. Previous studies showed that CCL2 is elevated in the cerebrospinal fluid of CM patients and linked to the development of brain pathology (6, 19). Thus, we hypothesize that the CCL2/CCR2 pathway modulates CNS inflammation and contributes to inflammatory pathology in CM.

Here, using a murine model of CM, we found that the CCR2 pathway drives fatal pathology even though it contributes to the fungal clearance. CCR2^−/−^ CM mice are largely protected from neurological deterioration and mortality. Improved CM outcomes in CCR2^−/−^ mice were marked by reduced neuronal cell death and restored gene expressions related to neurotransmission. Accumulation of CD11b^+^Ly6C^+^ IM, the major myeloid population infiltrating the brain during CM, depended almost entirely on CCR2 signaling. CCR2 modulates the accumulation and ultra-Th1 polarization of CD4^+^ T cells previously demonstrated to be pathological during CM. Our results suggest that therapeutic strategies targeting the CCR2 pathway could reduce neuronal pathology during severe CM.

## RESULTS

### CCR2 deficiency improves animal survival despite decreased fungal clearance during CM.

**FIG 1:**
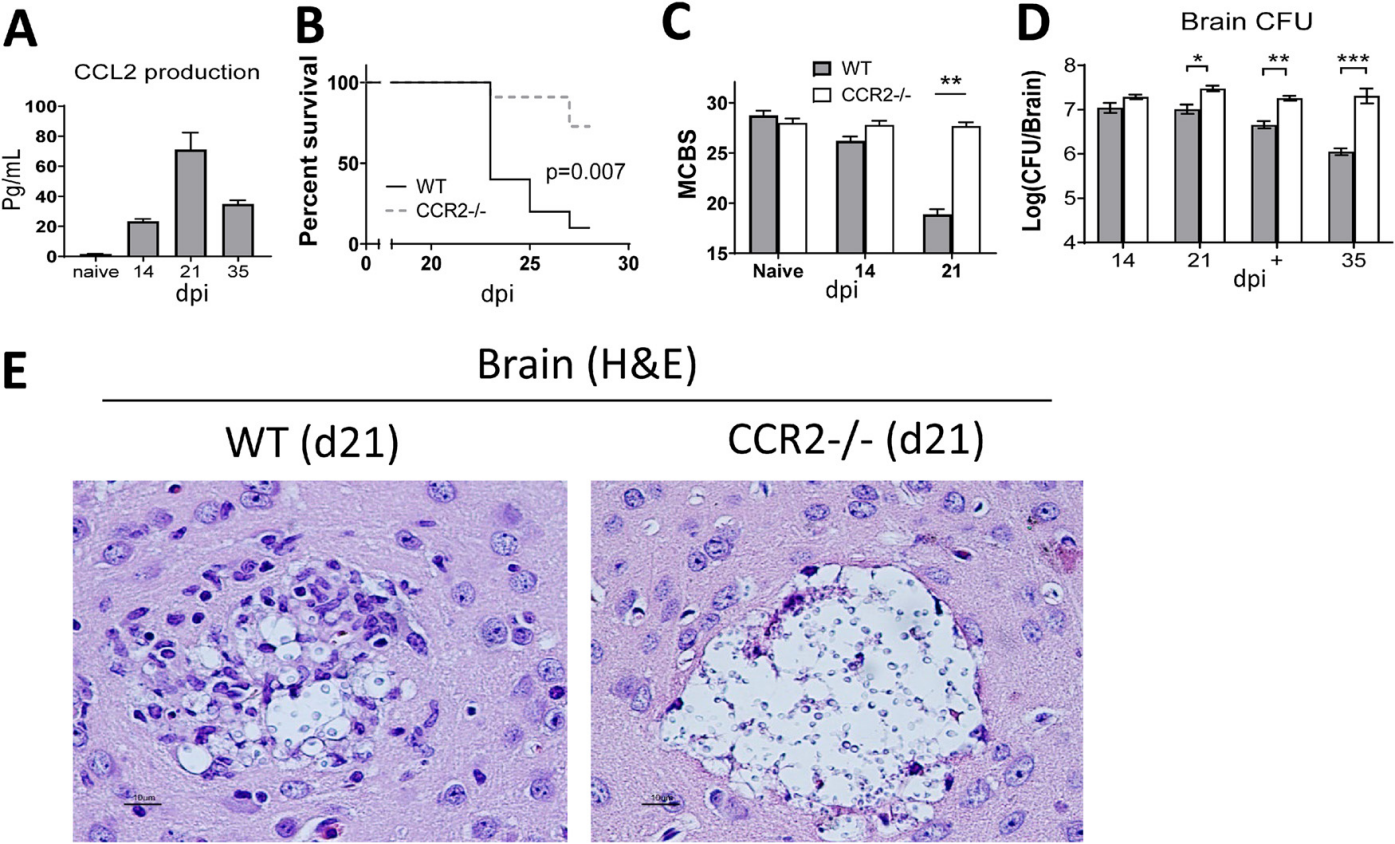
CCR2 drives immunopathology and mortality during CM. C57BL/6J mice were infected with 10^6^ CFU of C. neoformans 52D via retro-orbital intravenous inoculation. (A) Production of chemokine CCL2 in the brain homogenate at various time points was evaluated by CBA assay. (B) CCR2*^−/−^* mice showed prolonged survival and lower mortality than that of the WT-infected mice (*n* = 10). (C) MCBS scores of infected WT and CCR2*^−/−^* mice were compared to evaluate the overall health and neurological status. CCR2^−/−^ mice exhibited less-severe CM symptoms. (D) Brain fungal burdens were calculated on 14, 21, postdeath (+), and 35 dpi. CCR2 deficiency significantly impairs fungal control in the brain. We did not observe any significant difference in brain CFU, as well as CNS inflammation. (E) Brains from perfused WT and CCR2^−/−^ mice were paraffin-embedded, coronal sectioned, stained with hematoxylin and eosin (H&E), and imaged at (40×). Note that stained C. neoformans organisms occupy a relatively small area in the WT compared to that of CCR2-deficient mice and that C. neoformans are better contained and surrounded by the leukocyte infiltrate in WT mice but occupy a largely extracellular zone in CCR2^−/−^ mice. Data shown are the mean ± standard error of the mean (SEM) from an experiment representative of two independent experiments (*n* > 4). **, *P* < 0.01; ***, *P* < 0.001.

Elevated CCL2 in the cerebrospinal fluid of patients with HIV-associated CM predicts the risk of immune reconstitution inflammatory syndrome, linking overactivated CCL2/CCR2 axis with the development of inflammatory brain pathology (19). CCR2-dependent entry of IM into the CNS contributes to immunopathology in several neurological diseases (27–29). We thus evaluated the function of the CCL2-CCR2 axis in the pathological process of CM using our murine model, which reproduces human neuronal pathology associated with severe CM (12, 13). We found a significantly elevated level of CCL2 during CM, which began to increase at 14 days postinfection (dpi) and peaked at 21 dpi (Fig. 1A). The sharp increase in CCL2 expression marked the onset of mouse mortality post 21 dpi (Fig. 1B). About 80% of animals succumbed to infection between 21 and 30 dpi in wild-type (WT) mice. However, CCR2^−/−^ mice showed substantially improved survival, with only 25% mortality noted before 30 dpi (Fig. 1B). Unlike the WT mice, the infected CCR2^−/−^ mice did not develop severe neurological and behavioral defects, as quantified per murine coma and behavior scale (MCBS) (Fig. 1C). Strikingly, improved survival and behavioral readouts in CCR2^−/−^ mice were accompanied by a fungal load in the brain 2- to 3-fold higher than that in WT mice at 21 dpi and at the time of euthanasia (Fig. 1D). We did not observe significant differences in CFU, survival, or MCBS scores between animal sexes, and data from both sexes were included in the figures. Histological analysis showed more and larger cryptococcal clusters in the brains of CCR2^−/−^mice compared to those in the brains of WT mice at 21 dpi (Fig. 1E and see Fig. S1 in the supplemental material). However, cryptococcal microcysts in the WT mice were encircled by cellular inflammatory infiltrates, which were largely absent from the edges of cysts in CCR2^−/−^ brains (Fig. 1E). Thus, despite benefiting fungal clearance, the CCR2 axis contributed to severe CM symptoms and mortality that correlate with pathological evidence of brain inflammation.

10.1128/mBio.01076-21.1 FIG S1 Brains from perfused WT and CCR2^−/−^ mice were paraffin-embedded, coronal sectioned, and stained with hematoxylin and eosin (H&E). Note that C. neoformans formed more and larger cryptococcomas with fewer immune infiltrates in the CCR2^−/−^ mice than in WT mice. The data shown are results from an experiment representative of two independent experiments. Download FIG S1, PDF file, 0.2 MB. Copyright © 2021 Xu et al. 2021 Xu et al. https://creativecommons.org/licenses/by/4.0/ This content is distributed under the terms of the Creative Commons Attribution 4.0 International license.

### CCR2 deletion rescues neuronal cell dysfunction and apoptosis during CM.

**FIG 2:**
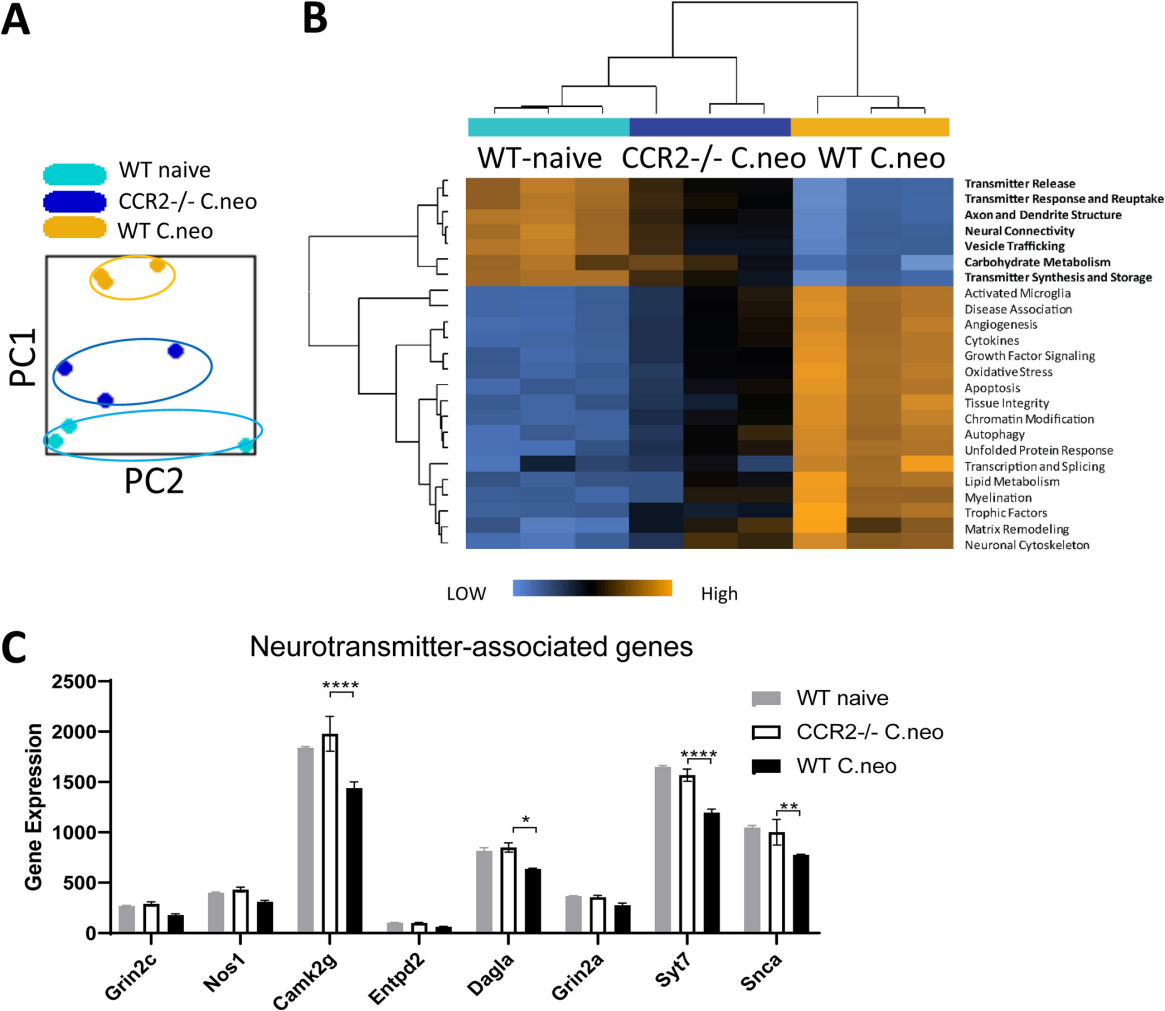
CCR2 contributes to dysregulated gene expression in neurotransmission pathways during CM. Gene transcripts of brain homogenates from naive and infected WT and infected CCR2*^−/−^* at 21 dpi were analyzed using a NanoString multiplex neuropathology panel. (A) Principal-component analysis of normalized read counts of the total 700 transcripts associated with neuropathology. Infected WT control and infected CCR2*^−/−^* mice formed separate clusters along with principal components 1 and 2. Data from NanoString shown are from one experiment with *n* = 3 samples per group, and each sample was pooled from 3 mice. (B) CCR2 deletion ameliorated the neurodegeneration and neuroinflammation pathway scores altered by CM in the WT mice. In particular, CCR2 deficiency preserved the overall pathways associated with neurotransmission and decreased expression of genes associated with neuroinflammation (activated microglia, cytokines), metabolism (autophagy, lipid metabolism, oxidative stress, transcription and splicing, unfolded protein response), and cell structure integrity (neural connectivity, neuronal cytoskeleton, tissue integrity). (C) Expression of selected genes associated with neurotransmission, *GRIN2C*, *NOS1*, *CAMK2G*, *ENTPD2*, *DAGLA*, *GRIN2A*, *SYT7*, and *SNCA*, was shown. Note that CM induced suppression of these genes in the brain of the WT mice but not in that of CCR2-deficient mice. Bars show mean ± SEM (*n* = 3), *, *P* < 0.05; **, *P* < 0.01; ****, *P* < 0.0001.

To establish molecular processes that accompany brain pathology during CM, we measured changes in mRNA transcripts using a NanoString neuropathology multiplex panel, which measures the expression of 700 genes associated with six fundamental pathways: neurotransmission, neuron-glia interactions, neuroplasticity, cell structure integrity, neuroinflammation, and metabolism. We measured the effect of CCR2 deletion on the development of these molecular signatures at 21 dpi, the peak of CNS inflammation during CM. Principal-component analysis of normalized gene transcript read counts consistently showed that the uninfected WT mice, infected WT mice, and infected CCR2^−/−^ mice formed separate clusters by principal components 1 and 2 (Fig. 2A). The CCR2^−/−^ mice with CM clustered between the infected and naive WT mice and closer to the uninfected mice. Analysis of specific pathway scores showed that CM resulted in a significant reduction of transcriptional signatures for physiological neurotransmission function (transmitter release, transmitter response and reuptake, transmitter synthesis and storage, vesicle trafficking) and carbohydrate metabolism relative to those of the uninfected brains (Fig. 2B). Concurrently, scores of pathways involved in neuroinflammation (activated microglia, cytokines), neurodegeneration and metabolic stress responses (autophagy, apoptosis, lipid metabolism, oxidative stress, transcription and splicing, unfolded protein response), and repair responses (neural connectivity, neuronal cytoskeleton, tissue integrity) increased significantly postinfection (Fig. 2B). CCR2 deletion largely prevented dysregulation in these pathways during CM, showing limited changes or resembling naive mice (Fig. 2B). Quantification of selected transcripts associated with neurotransmission, including *camk2g*, *dagla*, *syt7*, and *snca*, showed that CCR2 deficiency restored their suppressed expression (Fig. 2C). In addition to neuronal function genes, we analyzed the expression of transcriptional signatures of main resident cells known to be affected by and contribute to the inflammatory process within the CNS. CM significantly reduced oligodendrocyte and astrocyte but enriched microglia and endothelial cell transcriptional signatures in the WT mice (Fig. S2). In the infected CCR2^−/−^ mice, we observed that these effects of CM on cell signature scores were only partial (oligodendrocyte and microglia) or not present (astrocyte and endothelial cell) (Fig. S2), supporting that in the course of CM, CCR2 signaling affects the status of both neuronal and other CNS resident cells.

10.1128/mBio.01076-21.2 FIG S2 NanoString-detected, significant changes in cell-signature genes in the WT and CCR2^−/−^ mice with CM. Gene transcripts of brain homogenates from naive and infected WT and infected CCR2^−/−^ at 21 dpi were analyzed by NanoString multiplex neuropathology panel coupled with NanoString nSolver analysis. CM reduced oligodendrocyte and astrocyte significantly but enriched microglia and endothelial cell transcriptional signatures in the WT mice. In the infected CCR2^−/−^ mice, we observed that these effects of CM on cell signature gene scores were only partial (oligodendrocyte and microglia) or not present (astrocyte and endothelial cell). Download FIG S2, PDF file, 0.1 MB. Copyright © 2021 Xu et al. 2021 Xu et al. https://creativecommons.org/licenses/by/4.0/ This content is distributed under the terms of the Creative Commons Attribution 4.0 International license.

**FIG 3:**
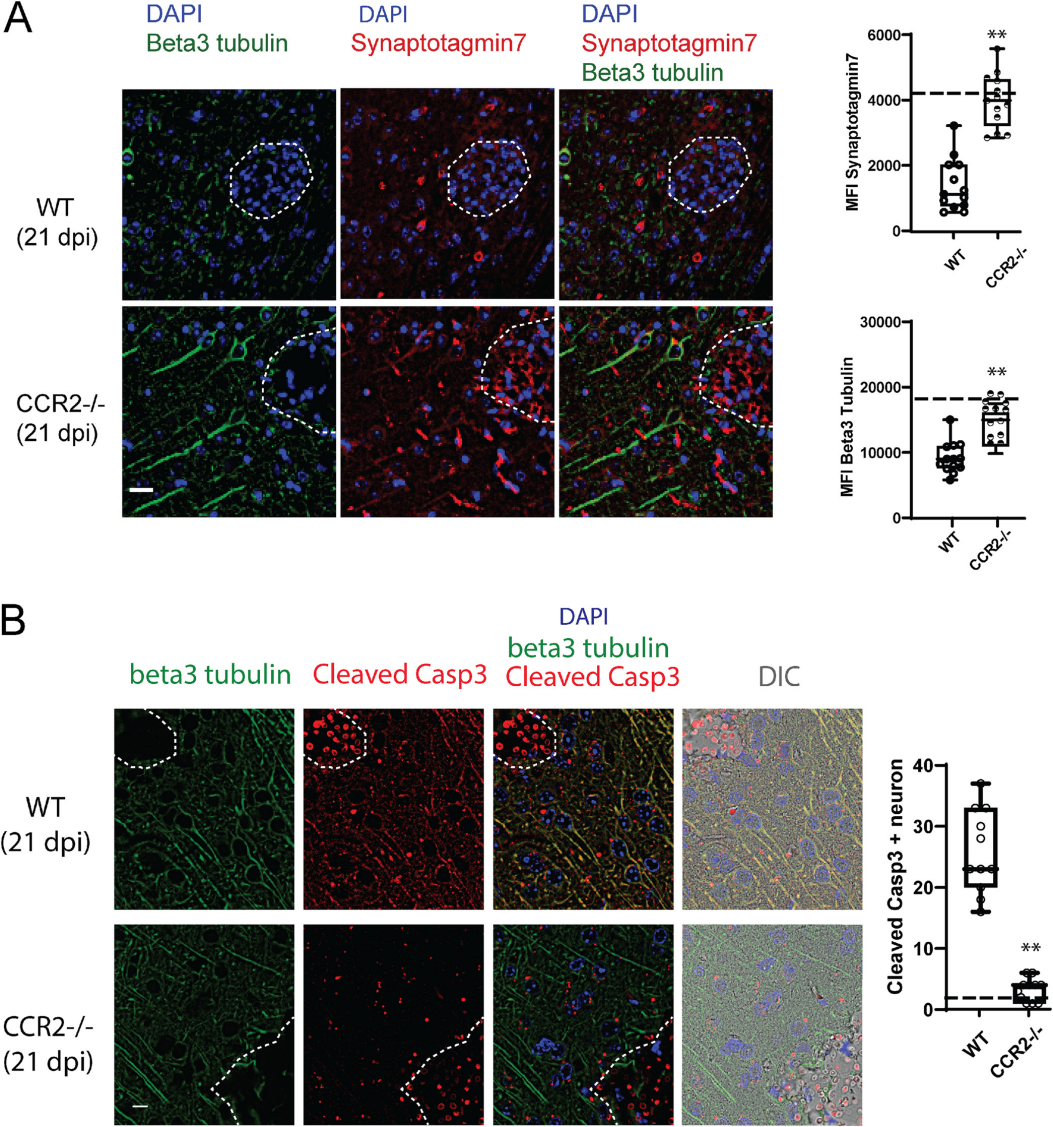
CCR2 pathway promotes regional defects in the production of neurotransmission proteins and neuronal cell apoptotic death. (A) Immunohistochemistry of the brain sections stained with antibodies to beta III tubulin (green) and synaptotagmin-7 (red). In the infected WT mice, neuronal damage was significant in the vicinity of the cryptococcal clusters, with weaker and less-organized beta III tubulin and synaptotagmin-7 signals. In the infected CCR2^−/−^ mice, neuronal damage in the vicinity of the cryptococcal clusters was limited. Dotted lines define the Cryptococcus lesions in the brain. The bar graphs on the right show the quantitation of the beta III tubulin and synaptotagmin-7 in the respective conditions, with dashed lines showing the corresponding signals from uninfected mice. Scale bar, 10 μm; magnification, 200×. (B) Immunohistochemistry of brain section stained with antibodies to beta III tubulin (green) and cleaved caspase-3 (red). In infected WT mice, cleaved caspase-3 and beta III tubulin overlay, indicative of dying neurons. In infected CCR2^−/−^ mice, signals of cleaved caspase-3 were minimal. Dotted lines define the Cryptococcus lesions in the brain. The bar graph on the right shows the quantitation of the cleaved caspase-3-positive neurons in the respective conditions, and dashed lines show the corresponding signals from uninfected mice. Scale bar, 5 μm; magnification, 600×. DIC, differential interference contrast. The data shown in A and B were from an experiment representative of two independent experiments (*n* = 2 mice per group), and at least 10 fields were examined for each sample.

Because of the very focal nature of the infection and inflammatory infiltrates in CM, the suppression of the neurotransmission gene expression measured in the global brain RNA may appear relatively modest (∼20% decrease) (Fig. 2C). However, these changes were highly significant, motivating our *in situ* quantitation of the selected markers at the perimeter of the individual cryptococcal lesions. We stained CNS sections from WT and CCR2^−/−^ mice for synaptic vesicle protein synaptotagmin-7 (Syt7), one of the top differentially expressed genes involved in neurotransmission pathways by our NanoString analysis. We found a uniform distribution of parallel neuronal cells (identified by β-III tubulin) with intracellular Syt7 expression, as well as extracellular Syt7, as evidence of postsynaptic vesicle release in both the uninfected WT and CCR2^−/−^ mice (Fig. S3). However, expressions of Syt7 and β-III tubulin at the perimeter of the cryptococcal microcysts were profoundly reduced in the infected WT mice relative to those in the infected CCR2^−/−^ mice (Fig. 3A), suggesting a severe defect in neurotransmission and loss of neuron cells. We further used cleaved caspase-3 (a marker of apoptosis) in β-III tubulin positive cells to measure potential neuronal death. In naive mice, β-III tubulin staining marked bundles of neurons in which cleaved caspase-3 signal was completely absent (Fig. S3). However, the cleaved caspase-3 fluorescence was high and colocalized with neurons at 21 dpi in the brains of the infected WT mice (Fig. 3B). Importantly, the level of the cleaved caspase-3 signal was minimal in the neurons of the infected CCR2^−/−^ mice (Fig. 3B). Together, we conclude that neurological symptoms and CNS pathology in CM manifest neuronal dysfunction and apoptotic death caused by the local inflammatory response, fueled by the CCR2 axis.

10.1128/mBio.01076-21.3 FIG S3 (A) Immunohistochemistry of the brain sections stained with antibodies to beta III tubulin (green) and synaptotagmin7 (red) in naive WT and CCR2^−/−^ mice sections show a uniform distribution of parallel neuronal axons. Synaptotagmin7 was expressed within the neurons or extracellularly due to postsynaptic release. Scale bar, 10 μm. (B) Immunohistochemistry of brain section stained with antibodies to beta III tubulin (green) and cleaved caspase3 (red) in naive WT and CCR2^−/−^ mice. The neuron morphology and distribution in naive mice were clear, and the overlay image is green due to the absence of cleaved caspase-3. Scale bar, 5 μm. The data shown are representative of results from a representative experiment of two independent experiments. Download FIG S3, PDF file, 0.4 MB. Copyright © 2021 Xu et al. 2021 Xu et al. https://creativecommons.org/licenses/by/4.0/ This content is distributed under the terms of the Creative Commons Attribution 4.0 International license.

### C. neoformans brain infection induces massive IM infiltration that depends on CCR2.

**FIG 4:**
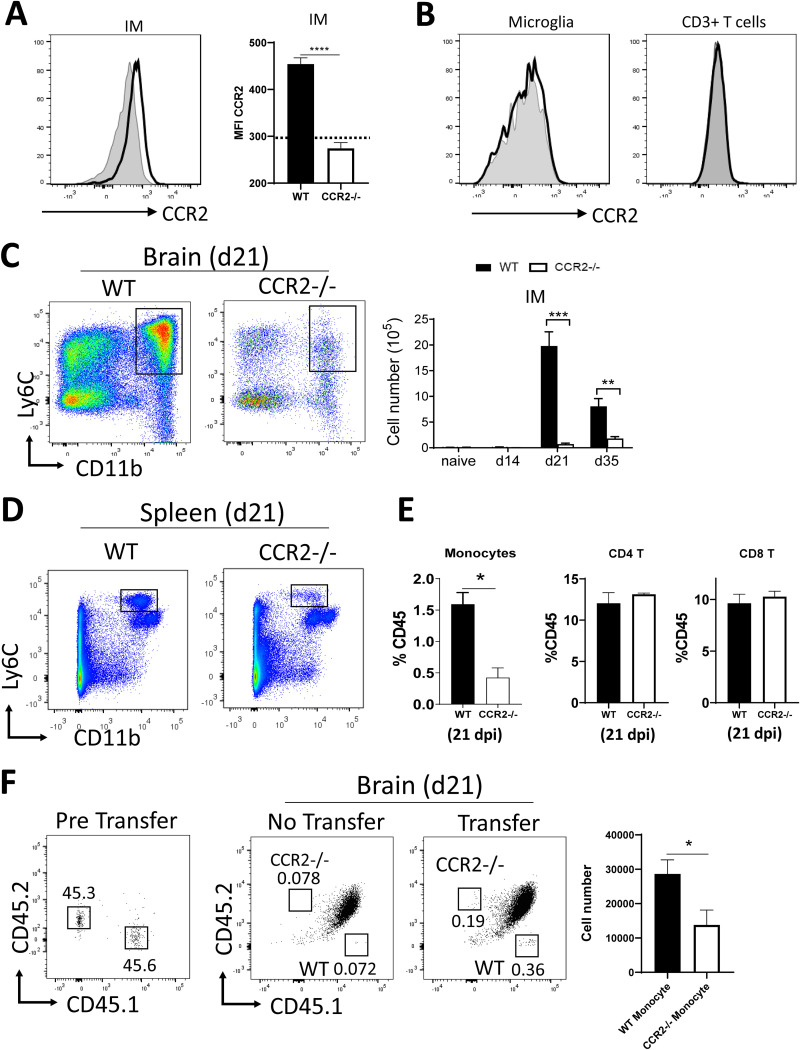
CCR2 pathway is essential for the accumulation of inflammatory monocytes (IM) in the C. neoformans*-*infected brain. Brain leukocytes from WT and CCR2^−/−^ mice were isolated and analyzed. (A and B) CCR2 is mainly expressed by IM but not microglia and T cells in the brain during CM. The gray area in the flow plot and dotted line in the bar graph represent the signal from isotype control. (C) Accumulation of Ly6C^+^CD11b^+^ IM in the infected brain at days 0, 14, 21, and 35 was evaluated. IM were absent until 14 dpi and showed peak accumulation on 21 dpi in WT mice. Note there was a profound reduction in the total number of IM in the brain of CCR2^−/−^ mice compared to that in the WT mice. (D and E) The frequencies of CD11b^+^Ly6C^+^ monocytes as well as CD4^+^ T cells and CD8^+^ T cells in spleens of WT and CCR2^−/−^ mice at 21 dpi. (F) Sorted monocytes from WT (CD45.1) or CCR2^−/−^ (CD45.2) mice were mixed at a 1:1 ratio and confirmed by flow cytometry (left panel) and then injected into WT (CD45.1 × CD45.2) recipients at 20 dpi. Frequency and the total numbers of WT and CCR2^−/−^ donor monocytes in the brain of recipient mice were analyzed by flow cytometry at 24 h posttransfer (right two panels). Representative flow cytometric dot plots are shown after CD11b^+^Ly6C^+^ IM gating. Numerical data or bar graphs showed mean ± SEM from an experiment representative of two to four independent experiments (*n* > 3). *, *P* < 0.05; **, *P* < 0.01; ***, *P* < 0.001.

Studies with pulmonary cryptococcal infection defined that CCR2 plays a critical role in the egress of monocytes from bone marrow, which in the lungs differentiate into effector IM eliminating C. neoformans (22, 23, 30). To assess the role of CCR2 in inflammatory cell recruitment during CM, we first evaluated the expression of CCR2 by brain leukocytes. We found that CCR2 was expressed on the surface of CD45^hi^CD11b^+^Ly6C^+^ IM (Fig. 4A and gating strategy in Fig. S4) but not microglia (CD45^low^CD11b^+^) or T cells (CD45^hi^CD3^+^) (Fig. 4B). CCR2 gene deletion abolished IM accumulation by 95% in the brain of CM mice at 21 dpi (Fig. 4C), suggesting an essential role of the CCR2 axis in IM accumulation in the brain during CM. Our fluorescence microscopy further showed that the accumulation of CD11b^+^ myeloid cells around the cryptococcal microcysts also depended on CCR2 signaling (Fig. S5). Systemically, infected CCR2^−/−^ mice showed a decreased frequency of splenic CD11b^+^Ly6C^+^ monocytes but not CD4^+^ T cells and CD8^+^ T cells in the spleens compared to those of WT mice (Fig. 4D and E), consistent with findings observed in our studies of cryptococcal lung infection, in which CCR2 was shown to mediate mainly Ly-6C^high^ monocyte egress from the bone marrow (22, 23).

10.1128/mBio.01076-21.4 FIG S4 Gating strategy of IM in the brain during CM by flow cytometry. CD45^hi^ infiltrating leukocytes were separated from CD45^low^ microglia (MG). Next, neutrophils were CD11b^+^Ly6G^+^ cells, and IM were identified as CD11b^+^Ly6C^hi^ cells in the remaining cells. Download FIG S4, PDF file, 0.2 MB. Copyright © 2021 Xu et al. 2021 Xu et al. https://creativecommons.org/licenses/by/4.0/ This content is distributed under the terms of the Creative Commons Attribution 4.0 International license.

10.1128/mBio.01076-21.5 FIG S5 CCR2-dependent IM accumulation around the cryptococcal lesions in the brain during CM. Immunohistochemistry of brain section stained with an antibody to CD11b (green). Note there was a profound reduction in the accumulation of IM around the cryptococcal lesions of CCR2^−/−^ mice compared to the WT mice. The data shown are results from a representative experiment of two independent experiments. Download FIG S5, PDF file, 0.1 MB. Copyright © 2021 Xu et al. 2021 Xu et al. https://creativecommons.org/licenses/by/4.0/ This content is distributed under the terms of the Creative Commons Attribution 4.0 International license.

To further determine if CCR2 was involved in monocyte trafficking from blood to CNS, we performed competitive adoptive transfers. Isolated monocytes from the bone marrow of WT CD45.1 and CCR2^−/−^ CD45.2 mice were cotransferred into WT CD45.1 × CD45.2 mice at 20 dpi. After 24 h posttransfer, we found that CCR2-deficient monocytes migrated into the brain of the recipient mice with 50% efficiency relative to that of the WT monocytes (Fig. 4F), demonstrating that CCR2 signaling contributes to monocyte recruitment from the blood into the brain. Taken together, we found that CCR2 is required for both monocyte egress from bone marrow and monocyte trafficking across the blood-brain barrier during CM.

### CCR2-gene deletion curtails neuroinflammation in the C. neoformans-infected brain.

**FIG 5:**
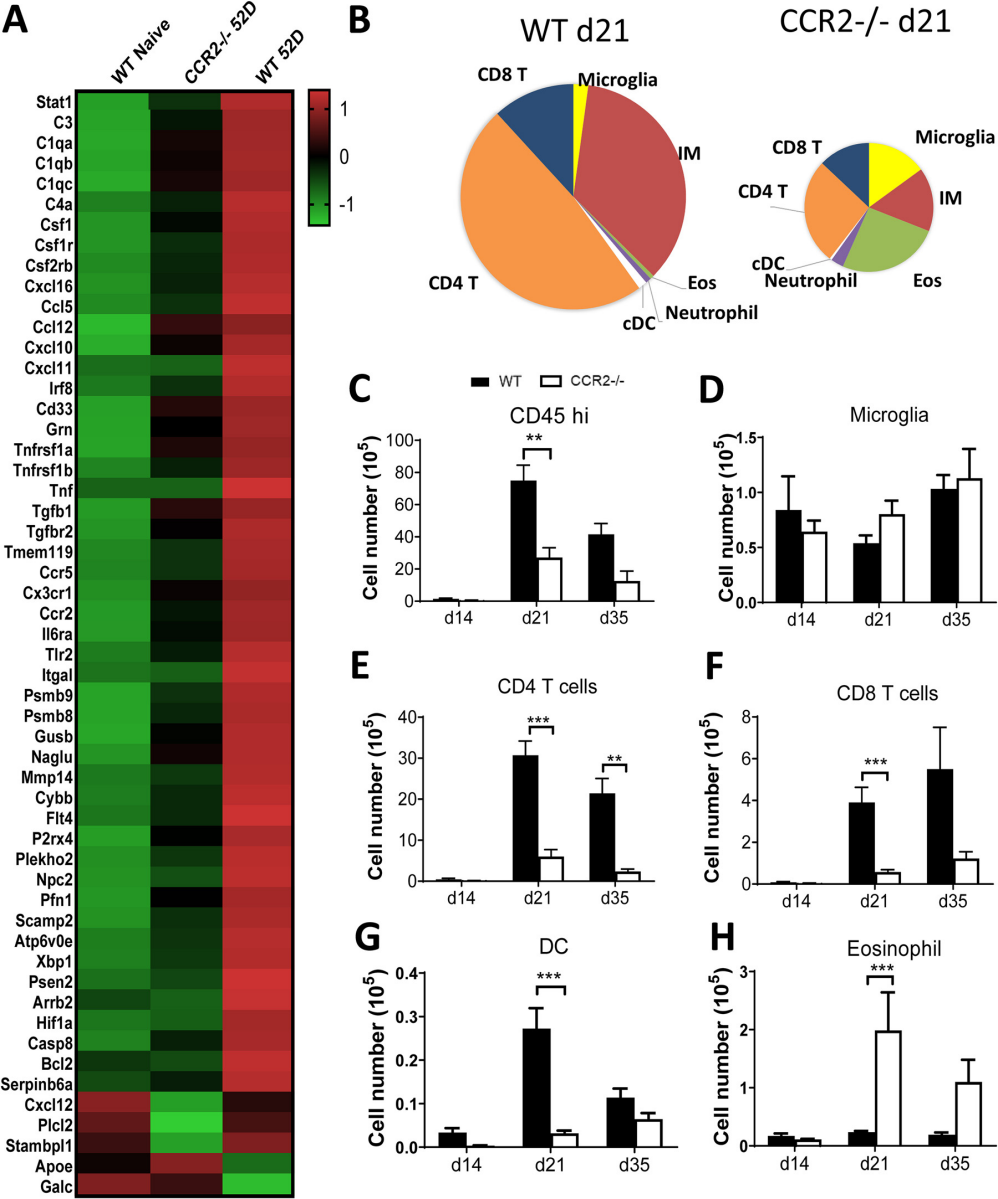
CCR2 modulates neuroinflammation during CM. (A) mRNA transcripts of immune-related genes in brain homogenate from naive WT, infected WT, and infected CCR2^−/−^ were analyzed using a NanoString multiplex neuropathology panel. The results show normalized gene expression relative to housekeeping genes in the NanoString multiplex panel. *n* = 3 per group. Note significant upregulation of inflammatory genes in the WT mice at 21 dpi and their minimal change in CCR2^−/−^ mice. Frequencies (B) and total numbers of brain-infiltrating CD45^hi^ leukocytes (C), microglia (D), CD4^+^ T cells (E), CD8^+^ T cells (F), DC (G), and eosinophils (H) in brains of WT and CCR2^−/−^ mice during CM. Mean ± SEM from an experiment representative of two to four independent experiments (*n* > 4). **, *P* < 0.01; ***, *P* < 0.001.

We next evaluated the impact of CCR2 signaling on neuroinflammation. CCR2 deletion profoundly altered the expression of neuroinflammation genes from the NanoString multiplex panel (Fig. 5A). Specifically, CCR2 gene deletion reduced expressions of Th1 signature genes (*stat1*, *cxcl10*, *tnf*, *tnfrsr1a*, and *tnfrsr1b*), chemokines, and cytokines, as well as their receptors involved in myeloid and T cell recruitment and activation (CCL5, CCL12, CXCL6, CXCL10, CXCL11, CSF1R, CCR5, IL4RA, IL6RA), and an array of complement proteins (C1QA, C1QB, C1QC, C3, C4A) (Fig. 5A). Since IM were the only cell subset showing surface expression of CCR2, our data strongly suggest that IM were either directly or indirectly responsible for the expression of these genes within the CNS. We further studied how CCR2-deletion affected leukocyte populations other than IM during CM. We found profound changes in leukocyte composition (Fig. 5B) and an overall decrease in global leukocyte accumulation in the CNS throughout the observed time course of CM (Fig. 5C). While we detected no changes in the total number of microglia, CCR2 deficiency impaired the recruitment of CD4^+^ T cells, CD8^+^ T cells, and dendritic cells (DCs) but enhanced eosinophil accumulation in the brain (Fig. 5C to H). These results demonstrate that the CCR2 axis, presumably via its effects on IM, profoundly reduced the overall magnitude of CNS inflammation and altered its characteristics during CM.

### CCR2 promotes pathological type 1 responses in the C. neoformans-infected brain.

**FIG 6:**
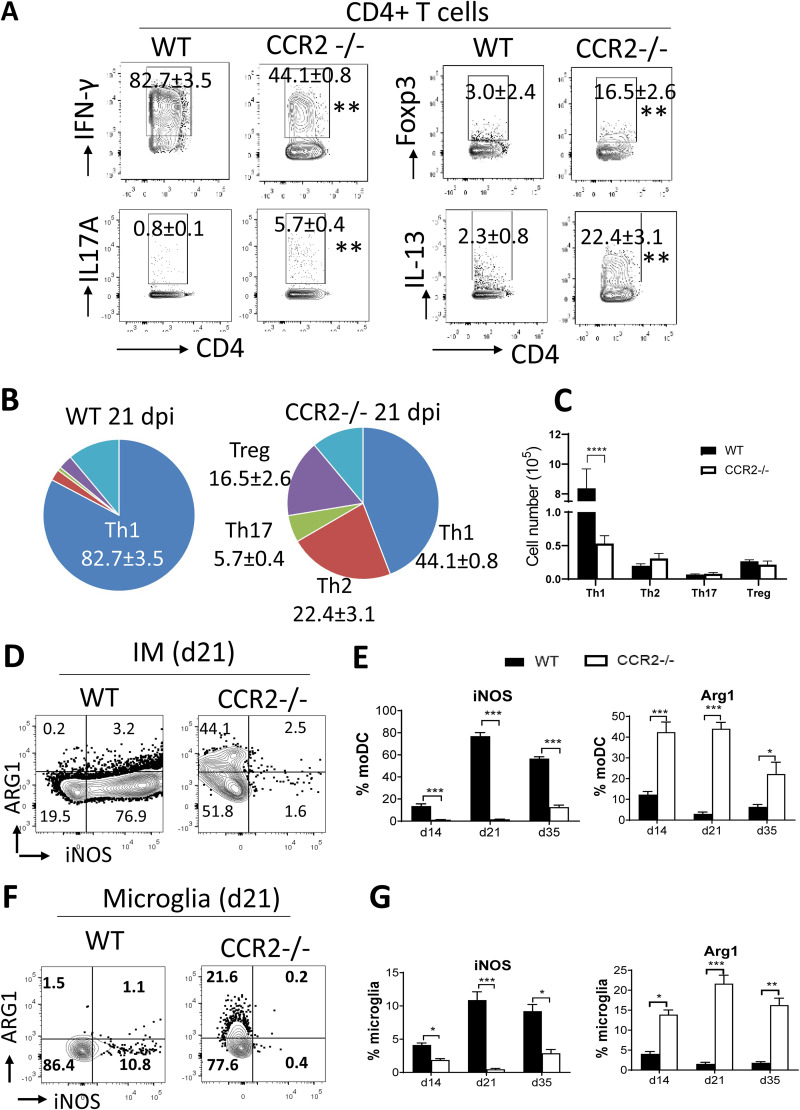
CCR2 contributes to pathological type 1 inflammation during C. neoformans brain infection. (A and B) Frequencies of CD4^+^ T cells producing IFN-γ, IL-17A, Foxp3, and IL-13 isolated from the brains of WT and CCR2^−/−^ mice 21 dpi show a shift in the immune polarization away from the ultra-Th1 response toward a more-balanced, mixed Th-phenotype in CCR2^−/−^ mice during CM. The pie charts show the ratio of different subsets of CD4^+^ T cells, Th1 (IFN-γ^+^), Th2 (IL-13^+^), Th17 (IL-17A^+^), Tregs (Foxp3^+^), and other cells without detectable cytokine productions. (C) Total cell numbers of CD4^+^ T cells subsets isolated from the brains of WT and CCR2^−/−^ mice at 21 dpi. (D and E) Expressions of iNOS and ARG1 by IM were analyzed in the brain of WT and CCR2^−/−^ mice by intracellular flow cytometry. Note that IM produce a high level of iNOS in infected WT but shift to express ARG1 in infected CCR2^−/−^ mice. (F and G) iNOS and ARG1 expression by microglia isolated from brains of WT and CCR2^−/−^ mice with CM. Mean ± SEM from an experiment representative of two to four independent experiments (*n* > 4). *, *P* < 0.05; **, *P* < 0.01; ***, *P* < 0.001.

Strong Th1 T cell polarization and elevated levels of interferon gamma (IFN-γ) in the cerebrospinal fluid of CM patients are associated with neurological deterioration (31, 32). Our previous study also revealed a pathological role of the ultra-Th1 polarization in our highly inflammatory model of murine CM (12). While links between CCR2 axis and T cell polarization have been well documented in the lungs during C. neoformans infection (23, 30, 33), this has not been explored in the CNS. We found that the frequencies of T cells producing IFN-γ and tumor necrosis factor alpha (TNF-α; Th1) were reduced in the brain of CCR2^−/−^ relative to those in the brain of WT mice (Fig. 6A and Fig. S6), while the frequencies of T cells producing interleukin 13 (IL-13; Th2), Foxp3 (Treg), and IL-17A (Th17) were increased (Fig. 6A and B). The absolute cell number of Th1 in the brain of CCR2^−/−^ mice decreased compared to that in the brain of WT mice, but the numbers of Th2, Th17, and Tregs were comparable in both mouse strains (Fig. 6C). Interestingly, even in the absence of CCR2, Th1 still remained the dominant polarization type in CD4^+^ T cells isolated from the CNS (Fig. 6B).

10.1128/mBio.01076-21.6 FIG S6 Frequencies of CD4^+^ T cells producing TNF-α isolated from the brains of WT and CCR2^−/−^ mice 21 dpi. Note that there was a profound reduction in the TNF-α producing CD4^+^ T cells in the brains of CCR2^−/−^ mice compared to those of WT mice, indicating a shift in the immune polarization resulting from CCR2 gene deletion. Data shown are the mean ± SEM from an experiment representative of two independent experiments (*n* > 4). **, *P* < 0.01. Download FIG S6, PDF file, 0.1 MB. Copyright © 2021 Xu et al. 2021 Xu et al. https://creativecommons.org/licenses/by/4.0/ This content is distributed under the terms of the Creative Commons Attribution 4.0 International license.

Th1 response stimulates myeloid cell classical activation, characterized by the expression of inducible nitric oxide synthase (iNOS) (25, 34). We found that more than 80% of IM in the WT mice produced high levels of iNOS at 21 dpi during CM (Fig. 6D and E), but only about 10% of microglia expressed iNOS (Fig. 6F and G). iNOS expression was ablated in CCR2^−/−^ mice in both IM and microglia (Fig. 6D). In contrast, the IM and microglia in the brain of CCR2^−/−^ mice expressed an increased level of arginase (ARG1) (Fig. 6D to G), an enzyme associated with an alternative-activation phenotype and nonfungicidal macrophages (35). Collectively, these data demonstrate that CCR2 plays a role in the development of the pathological ultra Th1 response and the subsequent strong M1 polarization within the CNS during CM.

## DISCUSSION

Evidence is accumulating to support the pivotal role of neuroinflammation in CM pathogenesis (36). In the current study, we demonstrate that (i) CM mortality and neurological deterioration are marked by neuronal cell death and dysfunction in the areas infiltrated by the inflammatory cells, (ii) CCR2 plays a critical role in driving both neuroinflammation and the lethal pathology despite promoting fungal clearance, (iii) while affecting multiple cell subsets, CCR2 was critically required for the recruitment of inflammatory monocytes (IM, CD45^hi^CD11b^+^Ly6C^+^) into the brain during CM, and (iv) CCR2 contributed to the accumulation of polarized Th1 CD4^+^ T cells, propelling the highly pathological ultra-Th1 immune response within the C. neoformans-infected brain. Together, our study demonstrates an essential role of the CCR2 signaling in modulating CNS inflammation and driving neuronal pathology.

Apart from the short-term neurological deterioration of CM patients (6), persistent neuropsychological deficits are increasingly recognized upon their recovery from the severe CM (6, 37–40). Even with successful fungal eradication, CM patients exhibit overall rates of neuropsychological deficits higher than standardized population norms (37–40). Elevated cerebrospinal fluid (CSF) neurofilament light chains (NFL), a marker of axonal damage, were detected in human patients and suggested ongoing neurological damage (6), but detailed molecular mechanisms of brain pathology during CM are lacking. Here, using a new approach to reduce the inflammatory response (by CCR2 deletion) in CM mice, in addition to our previous two approaches (T cell depletion and CXCR3 deletion) (12, 13), we clearly show that rather than the fungus itself, the host inflammatory response is responsible for the development of lethal CNS damage. For the first time, however, we performed a detailed analysis of transcriptional pathways linked to the CNS pathology using a NanoString panel designed for studies of neurological disorders. Compared to those of healthy brains, we found highly significant changes in global neurotransmission and neuroinflammation pathways. We found these alterations to be specifically concentrated around the leukocyte-infiltrated cryptococcal lesions using confocal microscopy. The most striking finding was that most of these pathological changes could be alleviated or even reversed upon the CCR2 deletion, which resulted in profoundly reduced inflammatory infiltration of the affected areas of the brain. These improvements at the molecular level corresponded to the improved behavioral readouts and the health status of the CCR2^−/−^ CM mice. While our study only begins to “zoom into” the specific groups of CNS genes that are altered during CM, future studies looking into the human homolog gene subset and correlating them with the clinical outcomes will help to identify the CM patients at the greatest risk of inflammatory brain damage. Stratifying patients into groups that require appropriate immunomodulatory therapies will guide rationalized treatment of CM patients and likely those with other CNS infections.

Immune responses in the delicate CNS need to be especially well balanced to control the infectious agents while also minimizing immune-mediated CNS damage (7, 36, 41, 42). CCL2 was previously identified as one of the major chemokines detected in the CSF of patients with HIV-associated CM and was associated with the risk for the development of life-threatening immune reconstitution inflammatory syndrome (IRIS) (19). Our study establishes a mechanistic link between CCL2/CCR2 pathway and brain pathology by demonstrating that CCR2 contributes to the development of neurological symptoms and mortality. Because targeting CCR2 or monocytes is a proposed strategy for targeted therapy in cancer, steatohepatitis, traumatic brain injury, Alzheimer’s disease, and multiple sclerosis (43–48), our work suggests that targeting this pathway might offer clinical benefits to patients with inflammatory CNS disease.

CCL2/CCR2 axis regulates the mobilization of monocytes from bone marrow to the inflammatory sites (21). CD14^hi^CD16-monocytes predominate in the CSF during CM (9), but their function and migration mechanisms are still not well understood. Here, we demonstrate that their murine equivalent, CCR2^+^CD11b^+^Ly6C^+^ IM, is the dominant myeloid cell subset in the brain of the cryptococcal-infected brains. The CCR2 deletion leads to reduced monocyte accumulation in the spleen, suggesting a defect in monocyte egress from the bone marrow as previously reported (21). Our adoptive transfer study showed that CCR2-positive monocytes migrate from the bloodstream into the CNS more efficiently than the CCR2-deficient monocytes. Therefore, CCR2 plays a role in mediating monocyte crossing the blood-brain barrier during CM, even though other pathways appear to be also involved at this stage. Together, both of these CCR2-dependent IM recruitment steps accounted for roughly 95% of their CNS recruitment at the peak of the inflammation time point in our CM model.

We previously showed that CXCR3^+^IFN-γ^+^ CD4^+^ T cells are required for disease pathology during CM (13). However, the T cells at the peripheral tissues require to be restimulated by the specific antigen-loaded APCs to perform their effector functions (49). In cryptococcal pulmonary infection, CCR2-dependent IM colocalization with CD4^+^ T cells in the bronchovascular infiltrates and promotes the local Th1 responses (23). Here, we demonstrate that CCR2-deletion leads to decreased accumulation and Th1-polarized CD4^+^ T cells in the brain but does not alter the magnitude of other Th-subsets during CM. Improved neurological symptoms in CCR2^−/−^ mice provide a clue that CCR2^+^ IM work hand-in-hand with CD4^+^ T cells to drive disease pathology. However, future studies are needed to determine whether IM work predominately as the recruiters and activators of the pathological T cells, as the pathological effectors directly inducing neuronal damage upon activation by the Th1 cells, or as contributors to both processes as described in other models of inflammatory CNS damage. The IM and T cell cross talk in jointly mediating pathology has been described in cerebral malaria and experimental autoimmune encephalomyelitis models, pointing out the critical role of T cell/IM interactions under different disease conditions (50–52). One hint regarding the possible direct pathological role of the IM is their strong classical activation status in the WT mice and CCR2 deficiency dramatically shifting their profile to an alternatively activated phenotype. The excessive activation of IM has been linked to tissue pathology in neurological diseases (17).

While the role of CCR2 in our CM model seems to be predominately linked to IM recruitment, CCR2 was reported to be expressed by other cells, such as Th17 and Treg in different disease models (53, 54). However, in our model, these mechanisms are unlikely to be at play since we find no detectable surface expression of CCR2 on cells other than IM, and CCR2 deletion has not affected recruitment of Th17 and Treg into the C. neoformans*-*infected brain.

Regarding translational value, our murine model uses an intravenous (i.v.) injection with a clinical isolate from a human patient (ATCC 24067, 52D), which is sufficiently virulent to establish a highly reproducible, controlled-onset mouse model of CM in the immunocompetent mice (12). As expected, these mice developed adaptive immune responses that aid fungal clearance but unfortunately with severe to lethal collateral damage. In human patients, most cryptococcal infections/expansion are predominantly caused by a weak immune system, which correlates with high brain fungal load and poor prognosis. However, many AIDS patients with very high CFU in their CSF exhibit relatively few symptoms and often have a better prognosis than those with CM without known immune impairment. In early time points of infection in the WT mice and through the observed time course in CCR2^−/−^ mice, we see high fungal burdens but very few neurological symptoms and relatively little inflammation. With the development of CNS inflammation in the WT mice, severe pathologies were developed in the CNS before we could observe any direct effects of the fungal pathogen. Thus, our model resembles outcomes in patients with severe refractory CM or those who developed c-IRIS. Our results may provide important insights into the immune pathogenesis and guidance for the development and use of pro- versus anti-inflammatory therapies to minimize CNS injury. Our results imply that too-rapid immune-boosting treatments may not be beneficial or could be harmful in some patients with CM and suggest that the CM patients’ inflammatory status (including levels of CCR2 ligands) within CNS should be monitored and appropriately “toned” therapeutically.

One limitation of our model is that the i.v.-injected strain grown on Sabouraud dextrose broth bypasses pulmonary defenses and *in vivo* changes that *Cryptococcus* normally undergoes before brain infection, and another is that the strain 52D falls in a midrange of virulence compared to many other clinical isolates that are more virulent. Whether and how these factors affect CNS inflammation and pathology will need to be investigated.

In summary, we found that CCR2 orchestrates lethal immune pathology despite its important role in fungal clearance during CM. CCR2 promotes the recruitment of IM into the brain and strongly modulates neuroinflammation during CM. Targeting the CCR2 pathway and IM may create new therapeutic opportunities as an adjunct therapy for CM, especially those with uncontrolled CNS inflammation.

## MATERIALS AND METHODS

### Mice.

Male and female C57BL/6J mice were obtained from Jackson Laboratories (Bar Harbor, ME). CCR2^−/−^ mice (55) backcrossed to C57BL/6J were housed under specific-pathogen-free conditions in the Animal Care Facility at the VA Ann Arbor Healthcare System. Mice were 8 to 12 weeks old at the time of infection and were humanely euthanized by CO_2_ inhalation at the time of data collection. All experiments were approved by the Veterans Affairs Institutional Animal Care and Use Committee under protocol 1408-004 and were performed following NIH guidelines and the Guide for the Care and Use of Laboratory Animals. Data presented were from both male and female mice, and no significant differences in CFU, MCBS, survival, and CNS cell accumulation between animal sexes were found during C. neoformans brain infection.

### C. neoformans.

ATCC 24067 (American Type Culture Collection, Manassas, VA), C. neoformans strain 52D was recovered from 10% glycerol frozen stocks and grown for 96 h at 37°C in Sabouraud dextrose broth (1% Neopeptone, 2% dextrose; Difco, Detroit, MI) on a shaker. The cultures were then centrifuged and the pellets were washed with phosphate-buffered saline (PBS). Cells were counted via hemocytometer and diluted to a concentration of 5 × 10^6^/ml just before infection. Mice were infected with 10^6^ organisms (in 200 μl PBS) via retro-orbital intravenous injection under inhaled isoflurane anesthesia. Serial dilutions of the C. neoformans suspension were plated on Sabouraud dextrose agar to confirm the number of viable fungi in the inoculum.

### Murine coma and behavioral scale.

A murine coma and behavioral scale (MCBS) was used to assess the overall physical and neurological condition of infected mice as described previously (12, 13). In brief, mice were scored using a scale of 0 to 3 for exploration, balance, gait, body posture, coat condition, grip strength, reflexes (body, neck, pinna, and footpad reflexes), and response to visual stimuli. Lower scores reflect more-pronounced symptoms.

### Fungal burdens.

The total fungal burden was qualified on a per organ basis. Samples of brains and spleens homogenate were serially diluted with distilled water. Aliquots of each sample (10 μl) were plated on Sabouraud dextrose in duplicates. The CFU of the samples were counted after 48 h.

### Brain leukocyte isolation.

Leukocytes in the brain were isolated as described previously (12). Briefly, mice were euthanized with CO_2_ and then perfused with PBS to remove circulating red blood cells and leukocytes from the brain. The brains were then aseptically removed, transferred to GentleMACs C tubes containing 5 ml of digest medium (RPMI 1640 with 5% fetal bovine serum [FBS], 25 mM HEPES, GlutaMAX, penicillin-streptomycin, nonessential amino acids, sodium pyruvate, and beta-mercaptoethanol, collagenase [50 μg/ml; Roche], and DNase [100 U/ml; Worthington]). The tissue was minced and then processed on a GentleMACs homogenizer (Miltenyi). The homogenate was washed with RPMI 1640 and filtered through a 70-μm cell strainer. A discontinuous 30%/70% Percoll (GE Healthcare) gradient was used to remove cell debris, myelin, and neurons, and then microglia and brain-infiltrating leukocytes (BIL) were recovered from the interface. Isolated cells were then washed twice with PBS. Total cell numbers were determined by counting live cells on a hemocytometer with trypan blue.

### Flow cytometry.

After isolation, cells were stained with fixable live/dead dye (Life Technologies), blocked with anti-CD16/32, and stained with antibodies for CD45 (30-F11), CD3 (145-2C11), CD4 (GK1.5), CD8 (53-6.7), CD11b (M1/70), CD11c (N418), Ly6C (HK1.4), F4/80 (BM8), CD64 (X54-5/7.1), XCR1 (ZET), SIRPα (P84), and/or major histocompatibility complex class II (MHCII, M5/114.15.2).

For intracellular iNOS and ARG1 production by myeloid cells, the cells were stained for extracellular markers and then fixed and permeabilized with 2% formaldehyde for 30 min. Intracellular staining for iNOS (CXNFT) and ARG1 (IC5868P, R&D SYSTEMS) was performed in the permeabilization buffer.

For intracellular cytokine production by T cells, the cells were stimulated for 6 h with phorbol myristate acetate and ionomycin in the presence of brefeldin A and monensin for the final 4 h. The cells were stained for extracellular markers and then fixed with fixation/permeabilization buffer, and intracellular staining for Foxp3 and IFN-γ was performed in permeabilization buffer. Fluorescence minus one (FMO) controls were used for all experiments. Data were collected on either an LSRII cytometer (BD) or an LSRFortessa (BD) and were analyzed using FlowJo (TreeStar).

### NanoString analysis.

Total RNA was isolated from the whole-brain homogenate using TRIzol (Life Technologies) and sent for NanoString nCounter analysis (NanoString Technologies) (56). Briefly, target RNA was labeled with a capture probe and a reporter probe specific to the genes of interest. After hybridization, the probe-target complexes were immobilized on an imaging surface and then scanned by a fluorescence microscope. Data analysis was performed on the nSolver analysis software according to the manufacturer’s instructions and built-in statistical analyses. Pathway scores were calculated as the first principal component of the pathway genes’ normalized expression. The software will orient the scores such that increased score corresponds with increased expression in a majority of the pathway genes. Scores of the cell type characteristic genes were analyzed using the default settings from the cell type profiling algorithm in the nSolver analysis software.

### Microscopy.

Sections were deparaffinized in xylene and gradually rehydrated by a gradient of alcohol to water. Antigen retrieval was carried out in citrate buffer on a boiling water bath for 15 min. Sections were blocked in 5% bovine serum albumin (BSA) in PBS. For the beta III tubulin and synaptotagmin-7 staining, sections were stained with a rabbit polyclonal beta III antibody (1:100, Abcam) and synaptotagmin-7 antibody (clone S275, Invitrogen) for 1 h at 37°C. Secondary antibody staining was done with goat anti-mouse Alexa 555 antibody and goat anti-rabbit Alexa 488 antibody (1:500, Invitrogen) for 45 min at 37°C. For the beta III tubulin and cleaved caspase-3 stainings, sections were then stained with primary antibody to beta III tubulin (TuJ1) and a rabbit antibody specific to cleaved caspase-3 (1:100, Sigma) for 1 h at 37°C. Sections were then washed in PBS and counterstained with a secondary goat anti-mouse antibody secondary Alexa 647 antibody and goat anti-rabbit Alexa 555 secondary antibody (1:500) for 45 min at 37°C. After that, the sections were washed in PBS for 30 min. The autofluorescence was quenched by Vector’s true view kit (Vector Laboratories) as per the manufacturer’s protocol before mounting with mounting medium. Samples were visualized using a KEYENCE microscope BZ-X800 series. Images were captured by the camera provided by the vendor by using a ×20 objective 0.45 numerical aperture (NA) Nikon. Images were deconvolved for enhancing the clarity of the images. Images were analyzed by ImageJ, and the numbers of damaged neurons were calculated by counting the numbers of cleaved caspase-3 positive neurons around the area infected by *Cryptococcus*. Areas that are 500 square microns around the cryptococcal lesions were analyzed. All the images were captured under the same settings.

### *In vivo* monocyte adoptive transfer.

Bone marrow from CD45.1 WT and CD45.2 CCR2^−/−^ mice were harvested as previously described (57, 58). Monocytes were isolated using EasySep mouse monocyte isolation kit (STEMCELL Technologies) according to the manufacturer’s instructions. Wild-type and CCR2-deficient cells were mixed (2.5 × 10^6^ each per mouse) and coinjected via retro-orbital intravenous injection in 200 μl of PBS into CD45.1 × CD45.2 mice at 20 dpi. The brains were harvested and brain leukocytes were isolated and analyzed by flow cytometry 24 h after adoptive transfer.

### Cytokine expression.

Cytokine levels were measured from the supernatants of whole-brain homogenate after centrifugation (600 × *g*, 5 min) using LegendPlex cytometric bead assays (BioLegend) according to the manufacturer’s instructions.

### Statistical analysis.

Statistical analysis was performed using GraphPad Prism version 6 software with Student’s *t* test or analysis of variance (ANOVA) plus Tukey’s *post hoc* test for multiple comparisons. Asterisks on figures (in graphs or in the corners of flow cytometry plots) indicate statistical significance as follows: *, *P* < 0.05; **, *P* < 0.01; ***, *P* < 0.001; ****, *P* < 0.0001.
