# Supplementary material for: CCR2 Signaling Promotes Brain Infiltration of Inflammatory Monocytes and Contributes to Neuropathology during Cryptococcal Meningoencephalitis
Source: mBio. 2021 Jul 27;12(4):e01076-21. doi: 10.1128/mBio.01076-21 (PMC8406332; doi:10.1128/mBio.01076-21)
Supplement: FIG S1 [file mbio.01076-21-sf001.pdf]

Fig S1

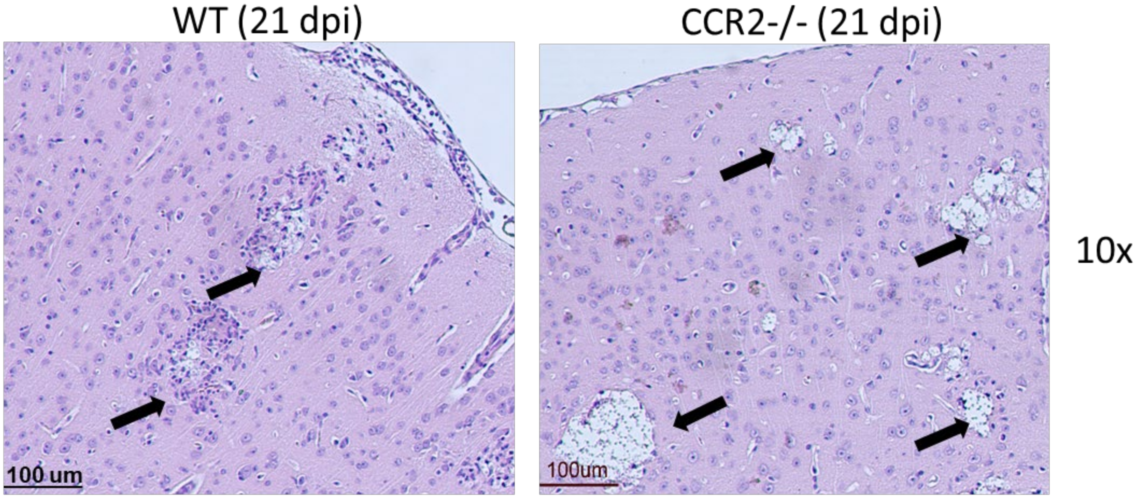

Fig S1. Brains from perfused WT and CCR2$^{-/-}$ mice were paraffin-embedded, coronal sectioned, and stained with hematoxylin and eosin (H&E). Note that *C. neoformans* formed more and larger cryptococcomas with fewer immune infiltrates in the CCR2$^{-/-}$ mice compared to WT mice. The data shown are results from a representative experiment of two independent experiments.
