# Supplementary material for: CCR2 Signaling Promotes Brain Infiltration of Inflammatory Monocytes and Contributes to Neuropathology during Cryptococcal Meningoencephalitis
Source: mBio. 2021 Jul 27;12(4):e01076-21. doi: 10.1128/mBio.01076-21 (PMC8406332; doi:10.1128/mBio.01076-21)
Supplement: FIG S2 [file mbio.01076-21-sf002.pdf]

Fig S2

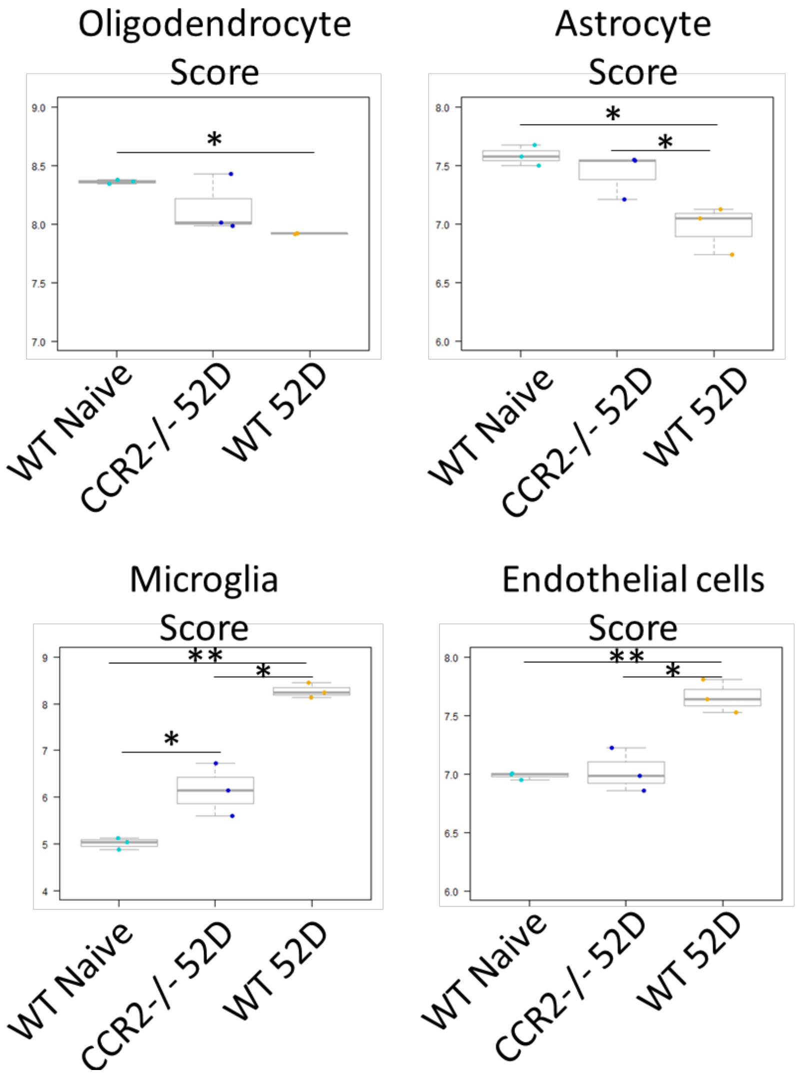

Fig S2 NanoString-detected, significant changes in cell-signature genes in the WT and $CCR2^{-/-}$ mice with CM. Gene transcripts of brain homogenates from naïve and infected WT and infected $CCR2^{-/-}$ at 21 dpi were analyzed by Nanostring multiplex neuropathology panel coupled with Nanostring nSolver analysis. CM significantly reduced oligodendrocyte and astrocyte but enriched microglia and endothelial cell transcriptional signatures in the WT mice. In the infected $CCR2^{-/-}$ mice, we observed that these effects of CM on cell signature gene scores were only partial (oligodendrocyte and microglia) or not present (astrocyte and endothelia).
