# Supplementary material for: CCR2 Signaling Promotes Brain Infiltration of Inflammatory Monocytes and Contributes to Neuropathology during Cryptococcal Meningoencephalitis
Source: mBio. 2021 Jul 27;12(4):e01076-21. doi: 10.1128/mBio.01076-21 (PMC8406332; doi:10.1128/mBio.01076-21)
Supplement: FIG S3 [file mbio.01076-21-sf003.pdf]

Fig S3

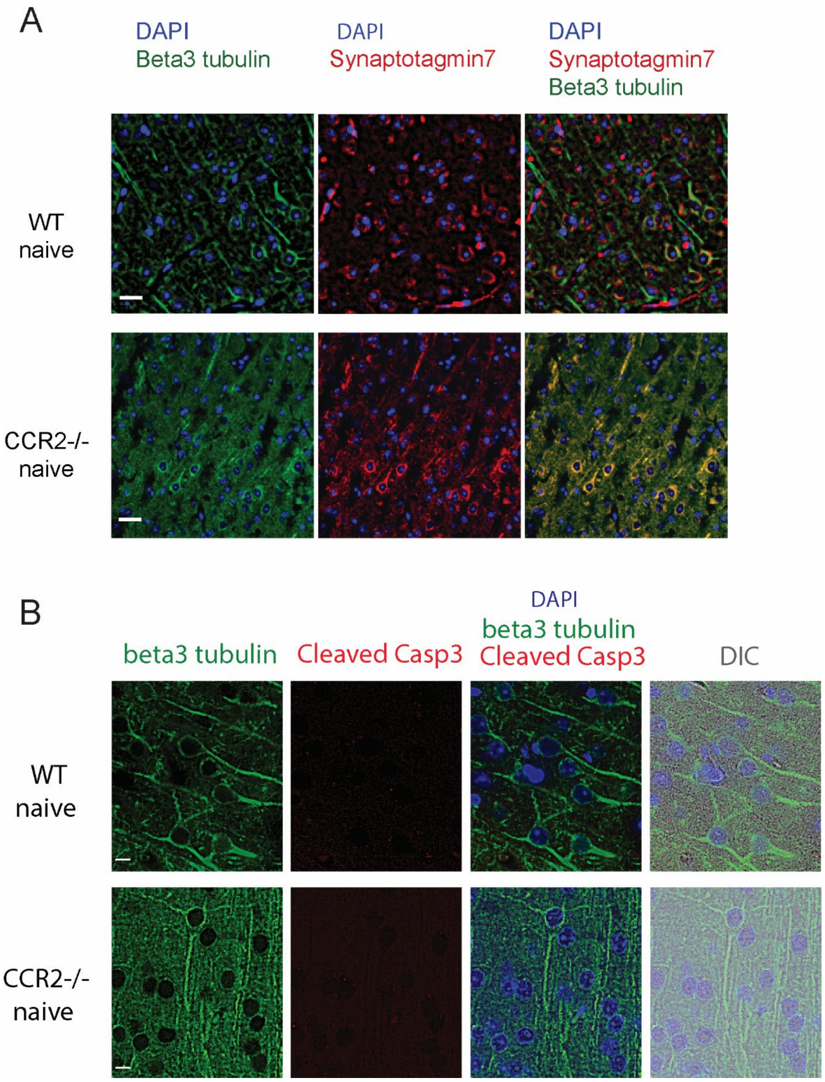

Fig S3. (A) Immunohistochemistry of the brain sections stained with antibodies to beta III tubulin (green) and synaptotagmin7 (red) in naïve WT and CCR2$^{-/-}$ mice sections show a uniform distribution of parallel neuronal axons. Synaptotagmin7 was expressed within the neurons or extracellularly due to postsynaptic release. (B) Immunohistochemistry of brain section stained with antibodies to beta III tubulin (green) and cleaved caspase3 (red) in naïve WT and CCR2$^{-/-}$ mice. The neuron morphology and distribution in naïve mice were clear, and the overlay image is green due to the absence of cleaved caspase 3. The data shown are results from a representative experiment of two independent experiments.
