# Supplementary material for: CCR2 Signaling Promotes Brain Infiltration of Inflammatory Monocytes and Contributes to Neuropathology during Cryptococcal Meningoencephalitis
Source: mBio. 2021 Jul 27;12(4):e01076-21. doi: 10.1128/mBio.01076-21 (PMC8406332; doi:10.1128/mBio.01076-21)
Supplement: FIG S4 [file mbio.01076-21-sf004.pdf]

Fig S4

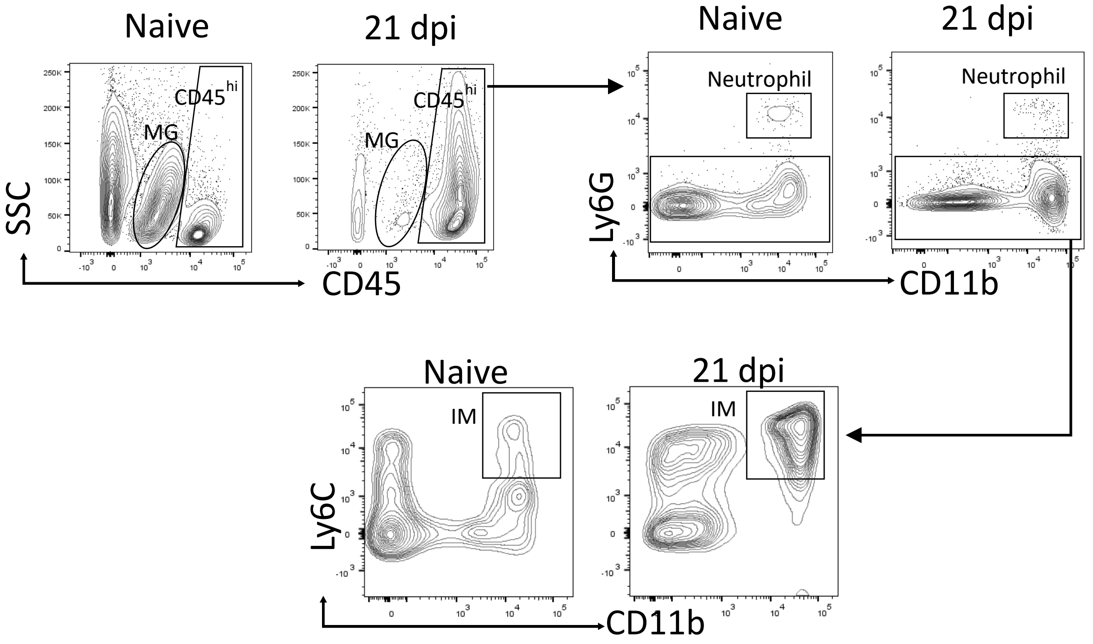

Fig S4. Gating strategy of IM in the brain during CM by flow cytometry. $CD45^{hi}$ infiltrating leukocytes were separated from $CD45^{low}$ microglia (MG). Next, neutrophils were CD11b+Ly6G+ cells, and IM were identified as $CD11b^{+}Ly6C^{hi}$ cells in the remaining cells.
