# Supplementary material for: CCR2 Signaling Promotes Brain Infiltration of Inflammatory Monocytes and Contributes to Neuropathology during Cryptococcal Meningoencephalitis
Source: mBio. 2021 Jul 27;12(4):e01076-21. doi: 10.1128/mBio.01076-21 (PMC8406332; doi:10.1128/mBio.01076-21)
Supplement: FIG S5 [file mbio.01076-21-sf005.pdf]

Fig S5.

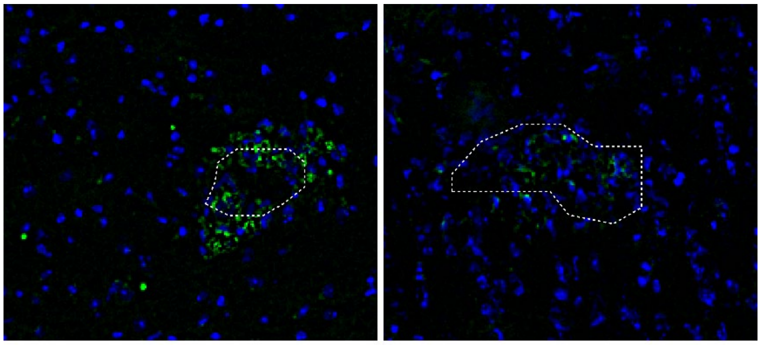

Fig S5. CCR2-dependent IM accumulation around the cryptococcal lesions in the brain during CM. Immunohistochemistry of brain section stained with an antibody to CD11b (green). Note there was a profound reduction in the accumulation of IM around the cryptococcal lesions of CCR2-/- mice compared to the WT mice. The data shown are results from a representative experiment of two independent experiments.
