# Supplementary material for: CCR2 Signaling Promotes Brain Infiltration of Inflammatory Monocytes and Contributes to Neuropathology during Cryptococcal Meningoencephalitis
Source: mBio. 2021 Jul 27;12(4):e01076-21. doi: 10.1128/mBio.01076-21 (PMC8406332; doi:10.1128/mBio.01076-21)
Supplement: FIG S6 [file mbio.01076-21-sf006.pdf]

Fig S6

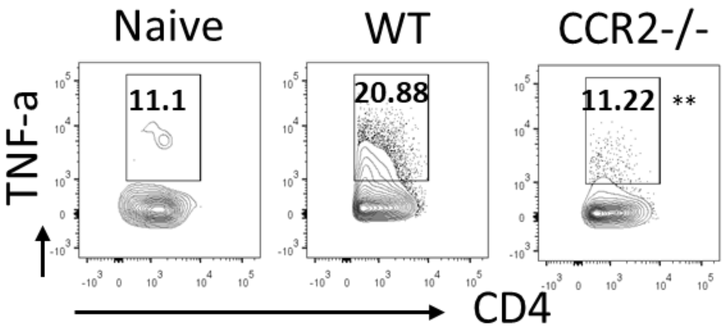

Fig S6. Frequencies of CD4+ T cells producing TNF-α isolated from the brains of WT and CCR2$^{-/-}$ mice 21 dpi. Note there was a profound reduction in the TNF-α producing CD4+ T cells in the brains of CCR2$^{-/-}$ mice compared to WT mice, indicating a shift in the immune polarization resulting from CCR2 gene deletion. Data shown are the mean ± SEM from a representative experiment of two independent experiments (n>4). **, P < 0.01.
